# Cell Cycle Arrest and Apoptosis Induction via Modulation of Mitochondrial Integrity by Bcl-2 Family Members and Caspase Dependence in* Dracaena cinnabari*-Treated H400 Human Oral Squamous Cell Carcinoma

**DOI:** 10.1155/2016/4904016

**Published:** 2016-03-30

**Authors:** Aied M. Alabsi, Kai Li Lim, Ian C. Paterson, Rola Ali-Saeed, Bushra A. Muharram

**Affiliations:** ^1^Department of Oral Biology and Biomedical Sciences, Faculty of Dentistry, University of Malaya, 50603 Kuala Lumpur, Malaysia; ^2^Oral Cancer Research and Coordinating Centre, Faculty of Dentistry, University of Malaya, 50603 Kuala Lumpur, Malaysia; ^3^Faculty of Bioresource, University Sultan Zainal Abidin, Terengganu, Malaysia; ^4^Faculty of Pharmacy, Sana'a University, Sana'a, Yemen

## Abstract

*Dracaena cinnabari* Balf.f. is a red resin endemic to Socotra Island, Yemen. Although there have been several reports on its therapeutic properties, information on its cytotoxicity and anticancer effects is very limited. This study utilized a bioassay-guided fractionation approach to determine the cytotoxic and apoptosis-inducing effects of* D. cinnabari* on human oral squamous cell carcinoma (OSCC). The cytotoxic effects of* D. cinnabari* crude extract were observed in a panel of OSCC cell lines and were most pronounced in H400. Only fractions DCc and DCd were active on H400 cells; subfractions DCc15 and DCd16 exhibited the greatest cytotoxicity against H400 cells and* D. cinnabari *inhibited cells proliferation in a time-dependent manner. This was achieved primarily via apoptosis where externalization of phospholipid phosphatidylserine was observed using DAPI/Annexin V fluorescence double staining mechanism studied through mitochondrial membrane potential assay cytochrome *c* enzyme-linked immunosorbent and caspases activities revealed depolarization of mitochondrial membrane potential (MMP) and significant activation of caspases 9 and 3/7, concomitant with S phase arrest. Apoptotic proteins array suggested that MMP was regulated by Bcl-2 proteins family as results demonstrated an upregulation of Bax, Bad, and Bid as well as downregulation of Bcl-2. Hence,* D. cinnabari* has the potential to be developed as an anticancer agent.

## 1. Introduction

Oral squamous cell carcinoma (OSCC) is the sixth common malignancy in the world [1], principally due to the widespread use of tobacco and alcohol [2]. Despite recent advances in surgical and radio/chemotherapy protocols, the prognosis for patients with OSCC remains poor, particularly for those with late stage disease [3]. The discovery of new anticancer agents from natural products offers a promising new approach for the treatment of cancer, as it is hoped they may reduce the burden of side effects [4].

Natural products serve as a platform for the design and synthesis for many important new commercialized drugs [5]. The discovery as well as evaluation of plant-derived anticancer agents encompasses many steps, starting with the authentication and extraction of the plant material, followed by the separation and isolation of the constituents of interest, characterization of the isolated compounds, and quantitative evaluation [6]. Bioassay-guided fractionation has been recognized as an important method in the attempt to isolate pure biologically active compound from natural sources. Each fraction produced is evaluated in a bioassay system and only active fractions are further fractionated [7].* Dracaena cinnabari*, also known as dragon's blood, is a deep red resin that has been used as a traditional medicine in different cultures across the world. Locals of the Moomy city in Socotra Island have used* D. cinnabari* as a sort of “cure-all” to treat general wound healing, diarrhea, fevers, dysentery diseases, and internal ulcers of mouth, throat, intestines, and stomach [8]. Also, Yemeni people have used* D. cinnabari* as a folk medicine to cure dysentery, diarrhea, hemorrhage, and external ulcers [9]. With the latest technology, several active compounds had been isolated from the resin of* D. cinnabari* and these compounds have been reported to possess a wide spectrum of therapeutic properties, including antioxidant activity [10] and antimicrobial activity [11]. Anticancer activity against human bladder carcinoma cells has been reported [12]; however, the anticancer effects of* D. cinnabari* have not been thoroughly investigated.

In the present study we have utilized a bioassay-guided fractionation approach to evaluate the cytotoxic and apoptosis-inducing effects of* D. cinnabari* on OSCC cells. Fractions were isolated which exhibited cytotoxic effects that were selective against malignant cells compared to normal cells and these effects were associated with the induction of apoptosis, a depolarization mitochondrial membrane potential, translocation of cytochrome *c* from mitochondria into cytosol in H400 cells, and the activation of caspase 9. The apoptosis through modulation on mitochondrial integrity associated with Bcl-2 family proteins as well as cell cycle arrest. These data highlight the potential of* D. cinnabari* as an anticancer agent and provide a guide for future efforts to develop more potent anticancer drugs.

## 2. Materials and Methods

### 2.1. Plant Materials

*D. cinnabari* was collected from the Island of Socotra, Yemen. The plant samples were identified and authenticated by the Environmental Protection Authority of Yemen; Ministry of Water and Environment, Republic of Yemen, gave permission to conduct the study on this plant (Ethic number 2012 ه ع ح س|١١٣).

### 2.2. Extraction and Isolation

The powdered resin of* D. cinnabari* (50 g) was macerated with methanol (MeOH) (3 × 500 mL) (Merck, Darmstadt, Germany). The resultant extract was filtered using Whatman No. 1 filter paper (Whatman, England) and dried under vacuum to yield 28.0 g of the extract. The stock solution* D. cinnabari* crude extract (10 mg/mL) was prepared by dissolving the extract in DMSO and was then stored at −20°C for future use.

### 2.3. Bioassay-Guided Isolation

The methanolic extract was fractionated using vacuum liquid chromatographic (Merck, Germany) flash column chromatography. The extract (9.0 g) was fractionated on silica gel type H using VLC (4.0 × 25 cm, 100 g). The extract was then eluted with a solvent gradient of hexane/ethyl acetate (10 : 0, 3 × 200 mL; 9 : 1, 3 × 200 mL; 8 : 2, 3 × 200 mL; 7 : 3, 3 × 200 mL; 6 : 4, 3 × 200 mL; 1 : 1, 3 × 200 mL; 4 : 6, 3 × 200 mL; 3 : 7, 3 × 200 mL; 2 : 8, 3 × 200 mL; 1 : 9, 1 × 200 mL) and ethyl acetate/MeOH (100 : 0, 1 × 200 mL; 9 : 1, 2 × 200 mL; 7 : 3, 1 × 200 mL; 1 : 1, 1 × 250 mL; 0 : 100, 1 × 250 mL). Similar eluents were pooled according to their liquid chromatography mass spectrometry (LC-MS) profile using Shimadzu UFLC-IT-TOFMS, into seven fractions, DCa-DCg. Each fraction was dried under vacuum before being used in cell based assays.

The active fractions (DCc and DCd) were then further fractionated using preparative high performance liquid chromatography (HPLC) (Gilson GX-281/322 system) using a Waters Novapak C_18_ column (40 × 100 mm, 6 *μ*m). Fraction DCc was eluted at a flow rate of 12 mL min^−1^ over 120 min. The gradient elution started at 30% solvent (A) (acetonitrile with 0.1% formic acid) and 70% solvent B (water with 0.1% formic acid) for 5 min. The gradient then changed as follows: linear gradient from 30% to 50% (A) over 43 min; 50–70% (A) in 33 min; 70–100% in 15 min; and finally an isocratic elution of 100% (A) over 24 min. Similar subfractions were pooled according to their LC-MS profile into 16 subfractions, DCc1–DCc16.

**Figure 1:**
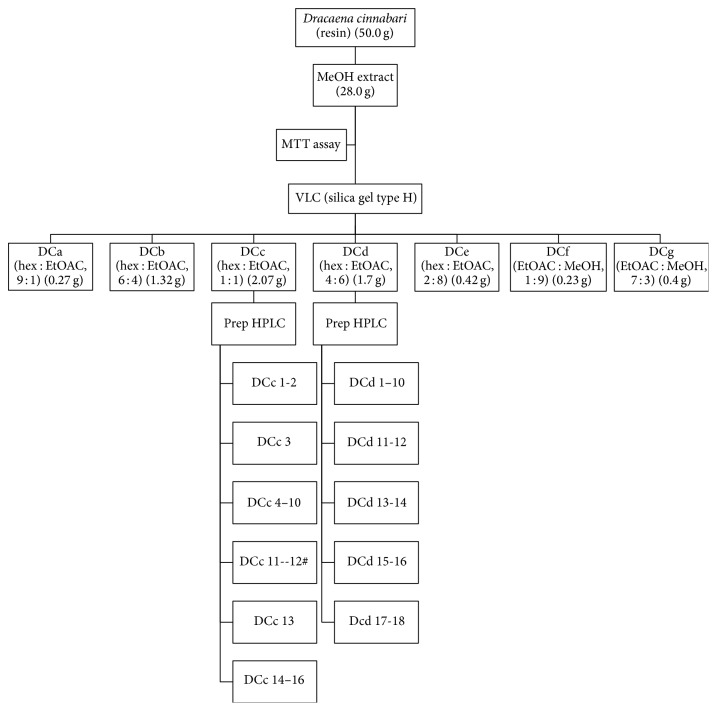
Flow chart of extraction procedure for* D. cinnabari*.

Fraction DCd was fractionated at a flow rate of 12 mL min^−1^ over 160 min. The linear gradient used was as follows: 20% A over 5 min; 20–30% from 5 to 48 min; then 30–50% A in 49 min followed by 50–100% A in 38 min; and finally an isocratic elution of 100% (A) over 38 min. Similar subfractions were combined according to their LC-MS profile into 18 subfractions, DCd1–DCd18. The extraction protocol is summarized in Figure 1.

### 2.4. Cell Culture

Six human oral squamous cell carcinoma (OSCC) cell lines and normal human oral fibroblast (NHOF) cells used in this study were obtained from Professor Ian Charles Paterson, Department of Oral Biology and Biomedical Sciences, Faculty of Dentistry, University of Malaya. The derivation and culture of the OSCC cell lines have been described previously [13]. Briefly, OSCC cell lines were cultured in T-75 cm^2^ culture flask (Corning, USA) containing DMEM/F-12 (Nacalai Tesque, Japan), supplemented with 10% (v/v) fetal bovine serum (FBS) (JR Scientific, USA). Normal human oral fibroblasts (NHOF) were cultured in DMEM (Gibco, USA) supplemented with 20% (v/v) FBS. Cells were grown at 37°C with 5% CO_2_ in a humidified atmosphere.

### 2.5. MTT Cytotoxicity and Proliferation Assay

The cytotoxicity of extract, fractions, and subfractions of* D. cinnabari* was tested using microculture tetrazolium test (MTT) assays, as previously described [14]. 1 × 10^4^ OSCC cells were seeded in 96-well microplate and cultured for 24 hours. Cells were treated with extract, fractions, and subfractions of* D. cinnabari* at a concentration range of 0 *μ*g/mL–30 *μ*g/mL for 72 hours. Untreated cells were regarded as negative controls, while cells treated with Cisplatin served as a positive control. Then, 20 *μ*L of freshly prepared MTT salt (5 mg/mL) was added into each well and incubated in dark for 4 hours. The media were removed and replaced with 150 *μ*L DMSO and the plate incubated for 10 minutes at 37°C to ensure all crystals were dissolved. Absorbance readings for the measurement and references wavelengths (575 nm and 650 nm, resp.) were determined using Tecan Infinite M200 Pro ELISA plate reader (Männedorf, Switzerland). Inhibitory concentration (IC_50_), concentration required to reduce cell viability by 50% as compared to the control cells, was determined.

In order to investigate whether the cytotoxic effects were specific to the cancer cells, selectivity indices (SI) for the studied compounds of* D. cinnabari* (DC extract, fractions DCc and DCd, and subfractions DCc15 and DCd16) were calculated. Selectivity index is defined as the ratio of cytotoxicity on normal human oral fibroblast cells to selected OSCC cells (SI = IC_50_ on NHOF cells/IC_50_ on OSCC cells) [15].

In experiments to measure proliferation rates, H400 cells were seeded in 96-well microplates as described above and treated with studied compounds of* D. cinnabari* (DC extract, fractions DCc and DCd, and subfractions DCc15 and DCd16) at IC_50_ concentrations for 24, 48, and 72 hours prior to MTT assay.

Percentage of cell viability was determined as follows:

(1) $$\begin{array}{c} \%\text{ viable cells}=\left(\;\frac{\text{OD sample}}{\text{OD control}}\;\right)\times 100, \end{array}$$

where OD is the optical density.

### 2.6. DAPI/Annexin V-FITC Fluorescence Double Staining

Mode of cell death either apoptosis or necrosis was determined through DAPI/Annexin V-FITC fluorescence double staining according to [16]. Briefly, 100 *μ*L of 1 × 10^5^ cells/mL of H400 cells was seeded in 96-well plate and incubated at 37°C, 5% CO_2_ overnight. Cells were then treated with studied compounds of* D. cinnabari* (DC extract, fractions DCc and DCd, and subfractions DCc15 and DCd16) at concentration of IC_50_ for 24 hours. Untreated cells served as negative controls.

After 24 hours of treatment, the medium in each well was discarded and 100 *μ*L of PBS was added. The 96-well plate was centrifuged at 3000 rpm for 1 minute and the supernatant was discarded. One hundred *μ*L, five *μ*L, and two *μ*L of 1x Annexin V-FITC binding buffer, Annexin V-FITC, and DAPI (100 *μ*g/mL) stains were added into each well, respectively, and incubation at room temperature in dark for 15 minutes was carried out. ImageXpress Micro Widefield High Content Screening System (Molecular Devices) was used to quantify and calculate the percentage of the apoptotic cells.

### 2.7. DNA Fragmentation

DNA fragmentation was determined through DNA laddering assay according to Ramasamy et al. (2012) [7]. Briefly, 1 × 10^5^ cells/mL of H400 cells were seeded and treated with DC extract, fractions DCc and DCd, and subfractions DCc15 and DCd16 of* D. cinnabari* at concentration of IC_50_ for 24, 48, and 72 hours. Plate was incubated at 37°C, 5% CO_2_ and untreated cells were used as negative control. After the treatment time point, cells were then trypsinized and washed with ice-cold PBS. Extraction of DNA was carried out using DNA extraction kit (Vivantis, USA) according to manufacturer's protocol. Purified DNA was eluted with 20 *μ*L of elution buffer and centrifugation at 5000 ×g for 1 minute was carried out. DNA was stored at −20°C. For detecting the DNA ladder pattern, the extracted DNA samples were run on 1.5% agarose gel in tris-acetic acid-EDTA (TAE) buffer. DNA band was observed and photographed using MultiDoc-It Digital Imaging System (UVP, Upland, CA, USA). Apoptosis induction was indicated by the appearance of DNA ladder fragments of approximately 180–200 bp multiples on the agarose gel.

### 2.8. Caspase Activities

Caspase 3/7, caspase 8, and caspase 9 activities in* D. cinnabari*-treated H400 cells were measured using caspase-Glo 3/7, caspase-Glo 8, and caspase-Glo 9 assay kits from Promega according to manufacturer protocol. Briefly, 100 *μ*L of 1 × 10^5^ cells/mL of H400 cells was seeded in a 96-well white plate and kept under 5% CO_2_ at 37°C for 24 hours. Cells were then treated with DC extract, fractions DCc and DCd, and subfractions DCc15 and DCd16 of* D. cinnabari* using IC_50_ concentration for 24 hours. Untreated cells were used as negative controls. MG132 inhibitor was added for caspase 8 and caspase 9 assays. Briefly, caspase-Glo buffer was thawed and equilibrated to room temperature prior to use. This buffer was then used to dissolve lyophilized caspase-Glo substrate and the mixture was vortexed to obtain a homogenous solution. Prior to the addition of caspase-Glo reagent, culture plate was equilibrated to room temperature. Then, 100 *μ*L of the reagent was added to each well and placed on orbital shaker for 30 seconds. The plate was then further incubated at room temperature for 2 hours in dark. The luminescence signal was then measured using Tecan Infinite M200 Pro ELISA plate reader (Männedorf, Switzerland).

### 2.9. Cytochrome *c* Detection

Quantification of cytochrome *c* was carried out using cytochrome *c* ELISA kit (Invitrogen, USA) according to the manufacturer's instructions. Briefly, 1 × 10^5^ cells/mL of H400 cells were seeded and treated with DC extract, fractions DCc and DCd, and subfractions DCc15 and DCd16 of* D. cinnabari* at concentration of IC_50_ for 24 hours. Untreated cells were used as negative control. After 24 hours of treatment, cells were harvested and washed twice with ice-cold PBS. The resulting cell pellets were stored at −80°C until they were used. Cell proteins were extracted using RIPA lyses buffer (Bio Basic Canada INC, Canada) supplemented with protease inhibitor, phosphatase inhibitor, and phenylmethylsulfonyl fluoride (PMSF) according to manufacturer's instructions. Cytochrome *c* in cell extracts was quantified using ELISA kit. Cytochrome *c* at concentration of 0–5 ng/mL was used as standard. Absorbance reading at 450 nm for samples and standard was measured using Tecan Infinite M200 Pro ELISA plate reader (Männedorf, Switzerland). Sample concentrations were determined using cytochrome *c* standard curve and expressed as ng per mL.

### 2.10. Mitochondrial Membrane Potential

Mitochondrial membrane potential was determined using JC-10 mitochondrial membrane potential assay kit (ABCAM, USA) according to manufacturer's instruction. Briefly, 90 *μ*L of 1 × 10^5^ cells/mL of H400 cells was seeded in a 96-well black colour plate and incubated at 5% CO_2_, 37°C for 24 hours. Treatment was carried out by adding 10 *μ*L of 10 times concentrated IC_50_ of DC extract, fractions DCc and DCd, and subfractions DCc15 and DCd16 of* D. cinnabari* for another 24 hours. Untreated cells were used as negative control. Fluorescence intensity at excitation/emission 490 nm/525 nm for green colour and 490 nm/590 nm for red colour were measured using Tecan Infinite M200 ELISA plate reader (Männedorf, Switzerland). Data was displayed in the ratio of green over red colour.

### 2.11. Human Apoptotic Proteins Array

Involvement of apoptotic proteins during apoptosis was determined through apoptosis array using Human Apoptosis Antibody Array kit (RayBiotech, GA, USA) according to the manufacturer's instructions. Briefly, 1 × 10^5^ cells/mL of H400 cells were seeded and treated with DC extract, fractions DCc and DCd, and subfractions DCc15 and DCd16 of* D. cinnabari* at concentration of IC_50_ for 72 hours. Untreated cells were used as negative control. After 72 hours of treatment, H400-treated cells were harvested and spun down at 2500 rpm, 4°C for 5 minutes. Cells were washed twice with ice-cold PBS. Centrifugation was carried out again at 2500 rpm, 4°C for 5 minutes and supernatant was discarded. Cell proteins were extracted and about 500 *μ*g of proteins from each sample was incubated with the human apoptosis array overnight. Chemiluminescence detections were carried out by scanning the membrane on Odyssey Fc Imaging System (LI-COR, USA).

### 2.12. Cell Cycle Analysis

Propidium Iodide Flow Cytometry Assay Kit (Abcam, USA) was used to determine the effect of DC extract, fractions DCc and DCd, and subfractions DCc15 and DCd16 of* D. cinnabari* on H400 cell cycle phases. Briefly, 1 × 10^5^ cells/mL of H400 cells were seeded into a 6-well plate and incubated at 37°C, 5% CO_2_ overnight. Cells were treated with studied compounds of* D. cinnabari* using IC_50_ concentration for 48 hours and 72 hours. After the treatment time points, trypsinization of the cells was carried out. Then, the cells were washed with PBS once and fixation of the cells was carried out using 66% ethanol. The fixed cells were stored at 4°C for at least 2 hours. After centrifugation at 500 ×g for 5 minutes, the cells were washed with PBS prior to staining. Two hundred *μ*L of staining solution composed of 1x PI and RNase was added to the cell suspension. The cells were incubated in dark at 37°C for 20 to 30 minutes and flow cytometry analysis was carried out using MASCQuant analyzer (Miltenyi Biotec, Germany).

### 2.13. Statistical Analysis

Data were expressed as mean ± SEM in triplicate. Statistical analysis was performed using one-way ANOVA (SPSS version 17). A *p* value <0.05 was considered statistically significant.

## 3. Results

### 3.1. Cytotoxic Activities of* D. cinnabari* Extract

The results of preliminary cytotoxicity screening of* D. cinnabari* crude extract towards six OSCC cell lines are summarized in Table 1. Cisplatin, a commonly used chemotherapeutic drug for the treatment of oral cancer in clinical based research, was used as positive control in these experiments. Based on the US National Cancer Institute (NCI) guidelines, a crude extract is generally considered to have* in vitro* cytotoxic activity if the IC_50_ value is ≤20 *μ*g/mL, while for a pure compound the IC_50_ value is ≤4 *μ*g/mL, following incubation between 48 and 72 hours [7, 17, 18]. The results demonstrated that* D. cinnabari* crude extract was cytotoxic towards all the six OSCC cell lines as the IC_50_ values ≤20 *μ*g/mL. The strongest cytotoxic effect was detected on H400 cells with an IC_50_ value of 5.9 *μ*g/mL. Therefore, H400 cells were selected as a targeted cell line for further screening with fractions and subfractions of* D. cinnabari*.

### 3.2. Bioassay-Guided Fractionation and Selectivity Index of* D. cinnabari*

Vacuum liquid chromatography (VLC) of* D. cinnabari* crude extract produced a total of seven fractions designated as DCa–DCg. The yield obtained and the cytotoxicity of fractions and subfractions against H400 cells are summarized in Table 2. Fraction DCd exhibited the greatest cytotoxic activity with an IC_50_ value of 3.1 *μ*g/mL, followed by fractions DCc, DCe, and DCa with IC_50_ values in the range 5.8 *μ*g/mL to 29.0 *μ*g/mL. For fractions DCb, DCf, and DCg, no cytotoxic effects can be observed as the IC_50_ >30 *μ*g/mL. Referring to a cut-off point of IC_50_ ≤10 *μ*g/mL as active fractions [19], fractions DCc and DCd were considered active towards H400 cells and they were subjected for second step of bioassay-guided fractionation.

A second step of bioassay-guided fractionation on fractions DCc and DCd yielded a total number of 16 subfractions (DCc1–DCc16) and 18 subfractions (DCd1–DCd18), respectively. Among the 16 subfractions of DCc, only 5 subfractions were cytotoxically active towards H400 cells with subfraction DCc15 showing the highest cytotoxic effect (IC_50_ values of 2.9 *μ*g/mL), whereas, among the 18 subfractions of DCd, only 7 subfractions were cytotoxically active towards H400 cells with subfraction DCd16 exhibiting the greatest cytotoxic effect (IC_50_ values of 2.9 *μ*g/mL). Throughout the cytotoxicity screening with bioassay-guided fractionation approach, crude extract (DC), fractions DCc and DCd, and subfractions DCc15 and DCd16 were selected as studied compounds for further study to investigate their effects on apoptosis.

Selectivity indices (SI) were calculated to determine whether the studied compounds of* D. cinnabari* exerted selective cytotoxic effects towards cancer cells compared to normal cells. In present study, SI value was determined through the cytotoxic activity on NHOF cells (Table 3). An SI value greater than 3 is considered to have high selectivity towards cancer cells [15]. Results demonstrated that subfraction DCc15 had IC_50_ values of 24 *μ*g/mL against NHOF cells with SI value of 8.28. No IC_50_ and SI values can be recorded for extract, fraction DCd, fraction DCc, and subfraction DCd16 as the IC_50_ values >30 *μ*g/mL. Thus, cytotoxicity activity of DC extract, fractions DCc and DCd, and subfractions DCc15 and DCd16 was considered selective towards H400 cancer cells.

### 3.3. *D. cinnabari* Inhibits H400 Cell Growth

**Figure 2:**
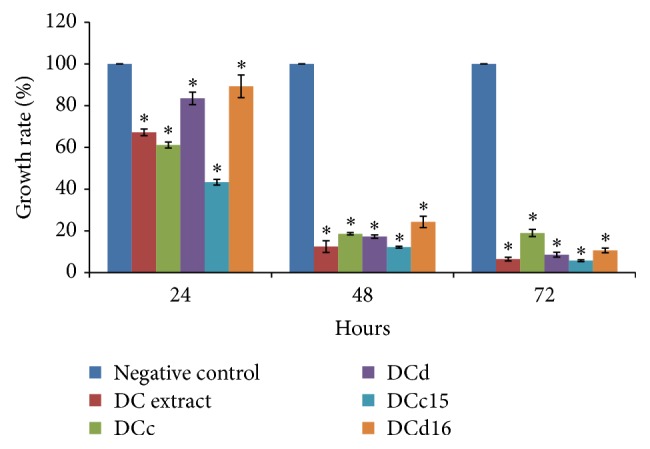
H400 cells proliferation upon treatment with DC extract, fractions DCc and DCd, and subfractions DCc15 and DCd16 of* D. cinnabari* at concentration of IC_50_ for 24, 48, and 72 hours. ^*∗*^Significant difference as compared to negative control (*p* < 0.05) when statistical analysis was performed using one-way ANOVA.

The effects of IC_50_ concentrations of DC extract, fractions DCc and DCd, subfractions DCc15 and DCd16 on the proliferation of H400 cells are shown in Figure 2. Results demonstrated H400 cells treated with* D. cinnabari* compounds inhibited H400 cells growth in a time-dependent manner as compared to untreated cells.

### 3.4. *D. cinnabari* Induces Externalization of Phospholipid Phosphatidylserine

**Figure 3:**
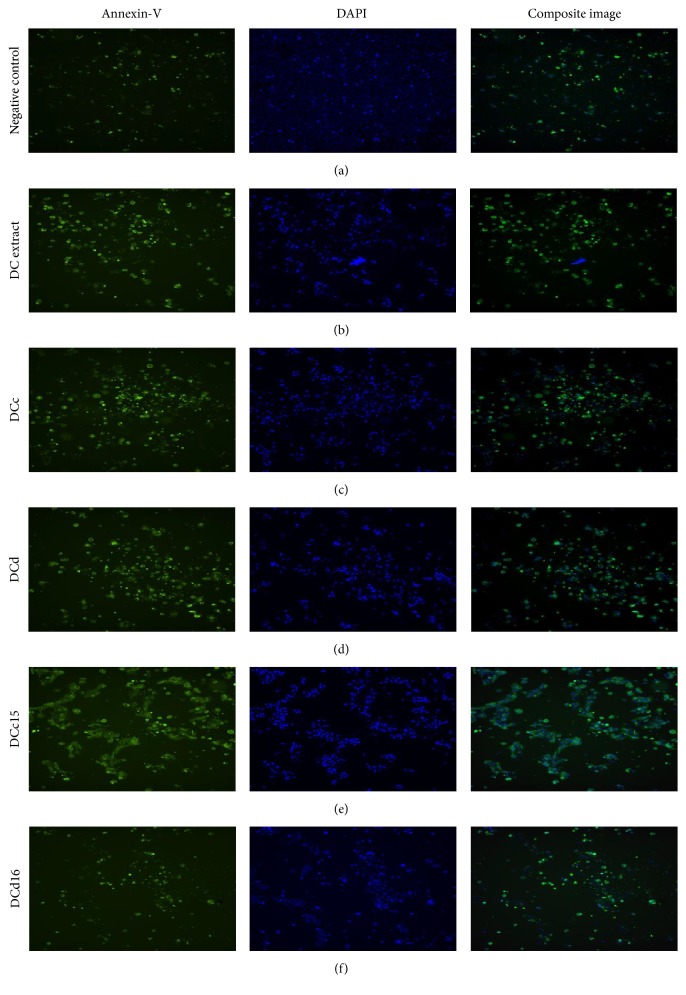
Morphological assessment of H400 cells treated with active DC extract, fractions DCc and DCd, and subfractions DCc15 and DCd16. H400 cells were treated at concentration of IC_50_ for 24 hours. Percentage of apoptotic cells was analyzed using ImageXpress Micro Widefield High Content Screening System after staining with DAPI/Annexin V-FITC (magnification 10x).

To investigate the mode of cell death, cells were stained with Annexin V-FITC and DAPI. As shown in Figure 3 only a small population of untreated H400 cells exhibited green and brighter blue fluorescence dye due to the Annexin V-FITC and DAPI binding. Most of the nuclei for untreated H400 cells stained weakly with DAPI and the cells showed clear evidence of cell-cell contact.

Most of the nuclei of H400 cells treated with DC extract, fractions DCc and DCd, and subfractions DCc15 and DCd16 were stained stronger with DAPI conjugated with Annexin V-FITC, indicating chromatin condensation and externalization of phospholipid phosphatidylserine. In addition, H400 cells exposed to these compounds showed a reduction in cell-cell contact as compared to untreated cells.  Table 4 shows the percentage of apoptotic cells increased from 13.81% (untreated cells) to a range of 41.99% to 85.69% following treatments of H400 cells with* D. cinnabari* extract and fractions. This revealed that the mode of cell death induced by DC extract, fractions DCc and DCd, and subfractions DCc15 and DCd16 was via apoptosis.

### 3.5. *D. cinnabari* Induces Internucleosomal DNA Fragmentation

Characteristic oligonucleosomal DNA fragments (ladders) were not observed in H400 cells treated with DC extract, fractions DCc and DCd, and subfractions DCc15 and DCd16 for the first 24 hours and 48 hours. However, at 72 hours, formation of DNA ladder was slightly observed on the 1.5% agarose gel. DNA isolated from untreated cells at 72 hours did not show any ladder formation. Hence, the efficient induction of apoptosis was observed at 72 hours in H400 cells treated with IC_50_ concentration of DC extract, fractions DCc and DCd, and subfractions DCc15 and DCd16.

### 3.6. *D. cinnabari* Induces Apoptosis through Intrinsic Caspase Pathway

**Figure 4:**
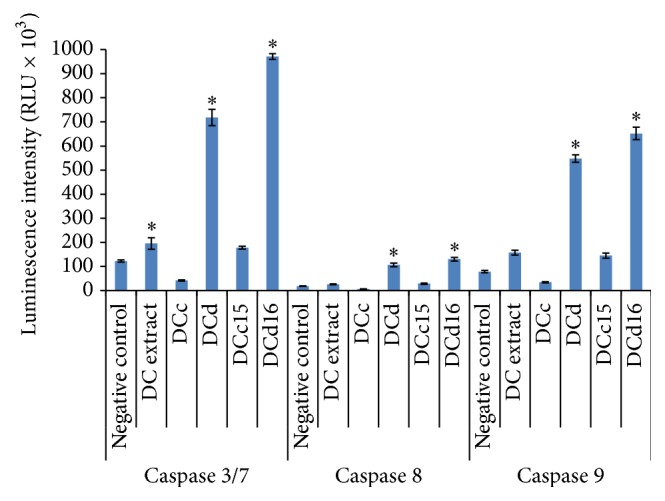
*D. cinnabari* induces apoptosis through intrinsic caspase pathway. Luminescence intensity of caspases 3/7, 8, and 9 in H400 cells treated with DC extract, fractions DCc and DCd, and subfractions DCc15 and DCd16 of* D. cinnabari* at concentration of IC_50_ for 24 hours. Untreated cells were used as negative control. Data was expressed as mean ± SEM (*n* = 3). ^*∗*^Significant difference as compared to negative control (*p* < 0.05) when statistical analysis was performed.

Apoptosis induction through either intrinsic or extrinsic pathways in H400 cells treated with studied compounds of* D. cinnabari* was examined by assessing the intracellular level of caspase 3/7, caspase 8, and caspase 9 activities (Figure 4). The results demonstrated that caspase 3/7, caspase 8, and caspase 9 were found to be enhanced upon the treatment with DC extract, fraction DCd, and subfractions DCc15 and DCd16 of* D. cinnabari*. The highest increment in RLU was observed in caspase 3/7 activity, followed by caspase 9 and caspase 8. This indicates that caspase 9 played a significant role as an initiator caspase to initiate the intrinsic apoptosis pathway following treatment with* D. cinnabari*.

### 3.7. Cytochrome *c* Detection

H400 cells treated with DC extract, fractions DCc and DCd, and subfraction DCd16 for 24 hours showed slight increase in cytochrome *c* compared to untreated cells. The cytochrome *c* concentration increased from 98.92 ± 0.93 ng/mL (control) to a range of 99.13 ± 0.40 ng/mL to 100.12 ± 1.38 ng/mL. However, H400 cells treated with subfraction DCc15 showed slight decrease in cytochrome *c* concentration (98.73 ± 0.81 ng/mL). Thus, studied compounds fractionated from* D. cinnabari* triggered the translocation of cytochrome *c* from mitochondrial membrane into cytosol in H400 cells.

### 3.8. *D. cinnabari* Induces Depolarization of Mitochondrial Membrane Potential (MMP)

**Figure 5:**
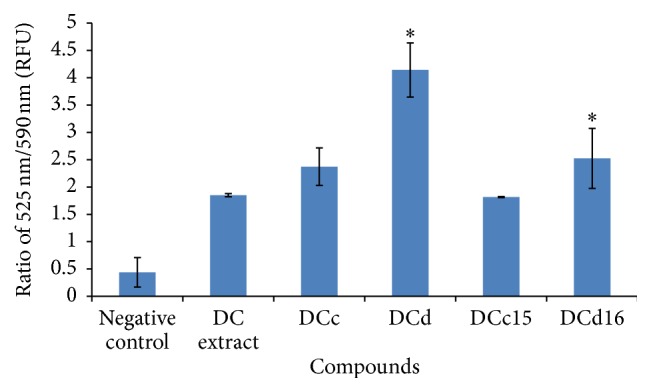
Changes in mitochondrial membrane potential in H400 cells treated with DC extract, fractions DCc and DCd, and subfractions DCc15 and DCd16 of* D. cinnabari.* H400 cells were treated at concentration of IC_50_ for 24 hours. Untreated cells were used as negative control. Data was expressed as mean ± SEM (*n* = 3). ^*∗*^Significant difference as compared to negative control (*p* < 0.05) when statistical analysis was performed.

Mitochondrial permeability transition is an important step in the induction of cellular apoptosis. Loss of mitochondrial potential (depolarization) is a classical evidence of apoptosis. In present study, H400 cells treated with DC extract, fractions DCc and DCd, and subfractions DCc15 and DCd16 of* D. cinnabari* for 24 hours showed increment in the ratio of green/red fluorescent intensity ranging from 1.81 RLU to 4.14 RLU compared to untreated cell (0.44 RLU). Mitochondrial membrane potential was reduced in H400 cells and the reduction was significant for fraction DCd and subfraction DCd16 (*p* < 0.05). This shows that the studied compounds of* D. cinnabari* induced depolarization of the mitochondrial membrane potential during apoptosis (Figure 5).

### 3.9. Apoptotic Proteins Array

**Figure 6:**
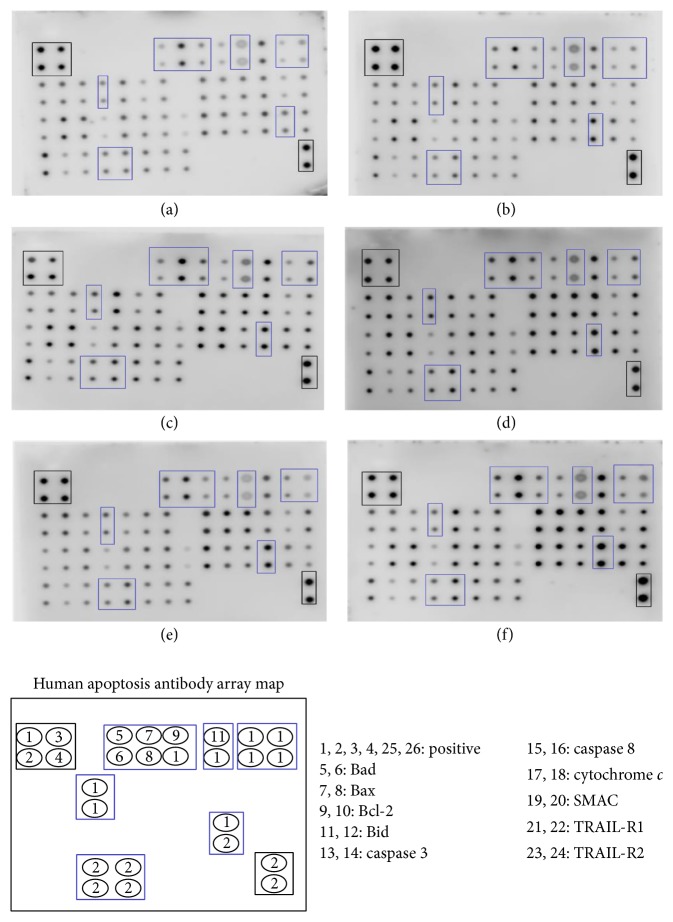
Human apoptotic proteins array for (a) untreated H400 cells; cells treated at concentration of IC_50_ with (b) DC extract, (c) fraction DCc, (d) fraction DCd, (e) subfraction DCc15, and (f) subfraction DCd16 for 72 hours.

Semiquantitative evaluations of apoptotic proteins in H400 cells treated with studied compounds of* D. cinnabari* for 72 hours at concentration of IC_50_ were determined through human apoptotic proteins array. The signal intensity for each antigen-specific antibody spot is proportional to the relative concentration of the antigen in the sample.  Figure 6 showed that the positive control signal on each array image has similar intensities. Hence, comparison of signal intensities for individual antigen-specific antibody spots among the array membranes can be used to determine differences in relative protein expression.

In present study, H400 cells treated with DC extract, fractions DCc and DCd, and subfractions DCc15 and DCd16 demonstrated upregulation of proapoptotic proteins such as Bax, Bad, and Bid and downregulation of antiapoptotic protein, Bcl-2. Besides, the elevation in the expressions of cytochrome *c* and SMAC in H400-treated cells was consistent with the earlier assay results which reported depolarization of the mitochondrial membrane potential leading to the translocation of these proteins into cytosol. Upregulation of both caspase 3 and caspase 8 further confirmed the previous results as demonstrated in caspases assay. In addition, there was an increment in TRAIL-R1 and TRAIL-R2 in H400 cells upon treatment with DC extract, fractions DCc and DCd, and subfractions DCc15 and DCd16.

### 3.10. *D. cinnabari* Causes Cell Cycle Arrest in S phase

**Figure 7:**
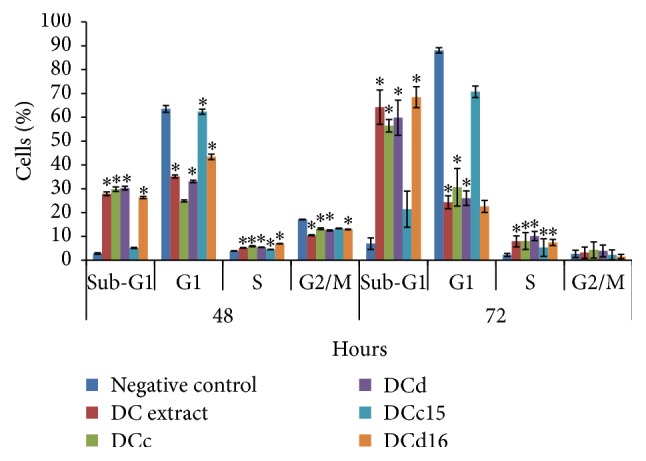
Flow cytometric analysis of cell cycle distribution in H400 cells. H400 cells were treated with DC extract, fractions DCc and DCd, and subfractions DCc15 and DCd16 of* D. cinnabari* at concentration of IC_50_ for 48 and 72 hours. Untreated cells were used as negative control. Data was shown as mean ± SEM. ^*∗*^Significant difference as compared to negative control (*p* < 0.05) when statistical analysis was performed.

The effect of* D. cinnabari* extract and fractions on the regulation of cell cycle was carried out using flow cytometry (Figure 7, Table 5). Compared with untreated H400 cells, the studied compounds of* D. cinnabari* resulted in a significant increase in H400 cells number in S phase upon 48 and 72 hours of treatment (*p* < 0.05). Additionally, the results demonstrated that the* D. cinnabari* extract and fractions showed a significant time-dependent increase in the proportion of sub-G1 phase, indicating apoptotic cells.

## 4. Discussion

The prognosis for patients diagnosed with OSCC remains very poor and new strategies for managing the disease are required. Screening natural products for anticancer effects represents one way to identify new therapeutic agents.* D. cinnabari* has been used to treat a variety of conditions by the people from the Moomy city in Socotra Island and in Yemen and it has been shown to possess a variety of biological properties, including antioxidant and antimicrobial effects [11, 20]. The present study has examined for the first time the anticancer effects of* D. cinnabari* against OSCC.

Based on NCI guidelines, a crude extract with IC_50_ value ≤20 *μ*g/mL and SI value >3 is considered to have strong cytotoxicity and is highly specific to cancer cells [7, 15, 17, 18, 21]. In the present study, a methanolic extract of* D. cinnabari* demonstrated marked cytotoxicity towards OSCC cell lines, results which are consistent with those of Al-Fatimi et al. (2005) [12] who showed anticancer effects on bladder cancer cells. Together, these data suggest that compounds from* D. cinnabari* may serve as promising new anticancer agents. Therefore, we carried out bioassay-guided fractionation on* D. cinnabari* to identify fractions that are capable of inhibiting the growth of OSCC cancer cells. To carry out the bioassay-guided fractionation, H400 cell lines were selected as a target cell line for further cytotoxic screening with fractions and subfractions of* D. cinnabari* as it was most sensitive to the effects of the crude extract (IC_50_ 5.9 *μ*g/mL). Fractionation of the methanolic extract of* D. cinnabari* yielded a total number of 7 fractions. To narrow the selection of active fractions that have the ultimate potential for biopharmaceutical use, a cut-off point of IC_50_ ≤10 *μ*g/mL was applied in this study. A similar approach was used by Siti Syarifah et al. (2011) [19], to identify cytotoxic fractions from* Cerbera odollam* using a bioassay-guided fractionation approach. In the present study, using this cut-off point, 2 out of 7 fractions were considered cytotoxically active towards H400 cells. These 2 active fractions, fractions DCc and DCd, were subjected for further fractionation using preparative high performance liquid chromatography (HPLC) to yield subfractions. A total number of 16 and 18 subfractions were isolated from fractions DCc and DCd, respectively. Subfractions DCc15 and DCd16 were the most active subfractions among the subfractions of DCc and DCd, respectively. NCI guidelines set a limit of IC_50_ ≤4 *μ*g/mL for a pure compound to be considered active [17]. Hence, both subfractions DCc15 and DCd16 were considered cytotoxically active towards H400 cells as the IC_50_ values ≤4 *μ*g/mL. The variation in the IC_50_ values between the fractions and subfractions of* D. cinnabari* was likely a result of different phytochemical constituents, the specific identity of which will form the basis of a future study.

An ideal anticancer drug should kill or incapacitate cancer cells without causing excessive damage to normal cells [22]. A selectivity index (SI) value is used as a reference point to determine whether the cytotoxic effect was specific towards cancer cells. SI values greater than 3 were considered to have high selectivity towards cancer cells [15]. Our results demonstrated that DC extract, fractions DCc and DCd, and subfractions DCc15 and DCd16 were specific towards H400 cells. These findings further highlight the potential of* D. cinnabari* to be developed as an anticancer drug.

There are two recognized modes of cell death, apoptosis and necrosis [23], and many studies have shown that the mechanism of action for anticancer drugs is based on the induction of apoptosis [7]. Externalization of phosphatidylserine (PS) from inner layer to outer layer of the cell membrane is the first indicator of apoptosis [24]. In present study, the presence of apoptotic cells was observed using Annexin V-FITC and DAPI fluorescence double staining. Annexin V-FITC binds to the externalization of PS and fluorescent green [16], whilst DAPI stain is used for counterstaining the nuclei of cells which fluorescence blue colour [25, 26]. The intensity of DAPI labeling represents the status of chromatin condensation; pale uniform DAPI staining indicated a normal and uncondensed chromatin, while brighter DAPI stain revealed a condensed chromatin which is a feature of apoptosis [27]. Our results suggested that the DC extract, fractions DCc and DCd, and subfractions DCc15 and DCd16 induced apoptosis in H400 cells, as the treated cells were stained with Annexin V-FITC and brighter DAPI stains which indicated externalization of PS conjugated and chromatin condensation, showing an increase in the percentage of apoptotic cells compared to untreated cells.

Important feature of cell apoptosis is the fragmentation of genomic DNA into integer multiples of 180–200 base pairs (bp) unit producing characteristic ladder on agarose gel electrophoresis. Activation of an endogenous endonuclease cleaves DNA in the linker region between histones on the chromosomes. Since the DNA wrapped around the histone comprises 180–200 bp, many of these intervals are characteristically observed and are commonly referred to as apoptotic ladder [7]. In present study, conventional agarose gel electrophoresis method was used in present study to analyze and observe the fragmented nuclei in H400 cells under ultraviolet light. Results demonstrated that no fragmentation of DNA was observed at 24, 48, and 72 hours of treatment with studied compounds of* D. cinnabari* on H400 cells. Fragmentation of DNA has been seen in many cell types and it is generally considered as the biochemical hallmark of apoptosis. Yet, it may be delayed, partial, or absent in some cell types [25]. Identification of apoptosis based on DNA laddering has led to an argument as some cells show apoptotic morphological changes in the absence of DNA fragmentation, making the formation of DNA ladders solely used as a marker of apoptosis unsuitable [28–30]. Degradation of DNA may be characteristic but not necessary for the sequence of events leading to apoptosis. There was no clear evidence presented to date that degradation of DNA would play a primary and causative role in apoptotic cell death. Besides, study by Gooch and Yee (1999) stated that DNA laddering represented a late event in the progression to apoptotic cell death and was not related to the ability of cancer cells to undergo apoptosis [30]. In addition, the absence or less expression of DNA fragmentation factor (DFF) in the cells could be a reason why the apoptotic ladder formation was not observed as this factor is essential for the apoptotic ladder formation [31]. In contrast, recent reports have described that electrophoresis technique in the detection of apoptosis was not very suitable for quantitative studies. High level of necrosis or the rapid onset of secondary necrosis may mask the ladders produced by low levels of apoptotic cell. Moreover, the possibility that induction of necrosis could generate ladder patterns on gel electrophoresis could not be ruled out [32]. These might explain the reasons why apoptotic ladder was not observed in H400 cells in this study.

Apoptosis can be an extrinsic pathway mediated via activation of death receptors or by an intrinsic mitochondria-mediated pathway [33]. Mitochondria play an important role in the regulation of apoptosis [34]. Fluorescent probes are tools frequently used to assess the mitochondrial function by monitoring the mitochondrial membrane potential (Δ*ψm*) [35]. In the present study we used JC-10 as a ratiometric fluorescent probe which was capable of entering selectively into mitochondria and changed reversibly its colour from green to orange as membrane potential increases. We demonstrated for the first time that the DC extract, fractions DCc and DCd, and subfractions DCc15 and DCd16 induced depolarization of mitochondrial membrane potential (MMP) in H400 cells. Therefore, one of the first steps by which the studied compounds of* D. cinnabari* trigger apoptosis is likely to be through changes in MMP. Depolarization of MMP causes the release of cytochrome *c* into cytosol which then leads to the activation of caspase 9 through the formation of apoptosome and subsequent cleavage of effectors caspase 3/7 [36]. In present study, treatment of H400 cells with the DC extract, fractions DCc and DCd, and subfractions DCc15 and DCd16 resulted in an increase in caspase 3/7 and caspase 9 activities, with a reduced and more variable effect on the activity of caspase 8. These data indicate that caspase 9 played a significant role as an initiator caspase to initiate the intrinsic apoptosis pathway. Taken together, our data strongly indicate that the studied compounds of* D. cinnabari* induced apoptosis via the intrinsic pathway by depolarising MMP and subsequent activation of downstream caspases.

In addition, changes in MMP cause the translocation of cytochrome *c* from the intermembrane space of the mitochondrial into cytosol [37]. Therefore, the intracellular level of cytochrome *c* in H400 cells was examined, using ELISA. Treated H400 cells showed a slight increment in cytochrome *c* concentration but the increment was not statistically different compared to untreated cells. This could be because of the cytochrome *c* that was just initiated to be released in H400 cells at 24 hours of treatment. Increasing the assay time to 48 and 72 hours of treatment might be necessary to detect more marked levels of cytochrome *c* translocation. The release of cytochrome *c* into the cytosol upon depolarization of mitochondrial membranes leads to the activation of caspase 9 through the formation of the apoptosome. Subsequent activation of the initiator caspase 9 causes the cleavage of effectors caspase 3/7 [38]. The significant increment of caspases 9 and 3/7 in present study provides evidence that the apoptosis induced by studied compounds of* D. cinnabari* in H400 cells was a caspase-dependent event.

There is growing evidence that Bcl-2 family proteins, which comprise proapoptotic and antiapoptotic proteins, are required in the modulation of mitochondrial integrity. The ratio of pro- to antiapoptotic proteins has been suggested to play an important role in determining commitment to cell death [39]. In present study, regulation of these apoptotic proteins was observed through apoptotic proteins array. Results demonstrated upregulation of proapoptotic proteins (Bad, Bax, and Bid) together with a downregulation of antiapoptotic protein (Bcl-2). Bad interacts with antiapoptotic proteins (Bcl-2) to ablate their prosurvival function which then allows the activation of Bax and Bak [40, 41]. An earlier study reported that activation of Bax and Bak caused the loss of mitochondrial membrane potential [42]. In addition, activation of Bax or Bak caused them to oligomerize in the outer mitochondrial membrane to generate large pores that released cytochrome *c* into cytosol [43]. Taken together, the results from the apoptotic proteins array which documented an upregulation of Bad and Bax and downregulation of Bcl-2 were concurred with the previous results that showed MMP depolarization as well as the release of cytochrome *c* and SMAC into cytosol of H400 cells treated with studied compounds of* D. cinnabari*.

Cell cycle, also known as cell division cycle, is a series of events which lead to cell division and duplication [44, 45]. Any dysregulation of the cell cycle machinery will lead to the development of cancers. The induction of cell cycle arrest at a specific checkpoint and thereby inducing apoptosis are a common mechanism for the cytotoxic effects of anticancer drugs [20]. It had been reported that many anticancer agents arrest cell cycle at G0/G1, S, or G2/M phase and then induced apoptosis cell death [46, 47]. Our data suggested that the studied compounds isolated from* D. cinnabari* arrested H400 cell at S phase after 48 and 72 hours of treatment. Interestingly, arrest of H400 cells at G2/M phase was observed only at 72 hours of treatment with DC extract and fractions DCc and DCd. Arrest of H400 cells in S and G2/M phases was accompanied with large accumulation of H400 cells in the sub-G1 phase. This proposed the relationship between H400 cells arrest at S and G2/M phases and apoptosis.

In summary, this study demonstrates for the first time that the DC extract, fractions DCc and DCd, and subfractions DCc15 and DCd16 of* D. cinnabari* showed selective cytotoxic towards H400 cells, compared to normal human oral fibroblast (NHOF) cells. Furthermore, these compounds were able to arrest H400 cells in S phase and G2/M phase and inhibited the proliferation of H400 cells. Besides, studied compounds of* D. cinnabari* induced apoptosis through the depolarization of mitochondrial membrane potential by downregulation of Bcl-2 and upregulation of Bax, Bad, and Bid, which trigger the translocation of cytochrome *c* and SMAC into cytosol of H400 cells. It is then followed by the activation of initiator caspase 9 and executioner caspase 3/7 which lead to the fragmentation of DNA. This form of apoptosis is associated with the extrinsic pathway through the activation of caspase 8 and Bid cleavage. In conclusion, our results highlight the potential of* D. cinnabari* to be developed as an anticancer agent.

## Figures and Tables

**Table 1:** Cytotoxicity (IC_50_) of *D. cinnabari* crude extract and Cisplatin on OSCC cell lines.

Cell lines	IC_50_ ± SEM (*μ*g/mL)^a^	
	*D. cinnabari* extract	Cisplatin
H400	5.9 ± 0.35	4.1 ± 0.55
H103	6.0 ± 0.08	0.8 ± 0.12
H376	7.2 ± 0.16	0.6 ± 0.03
H357	9.0 ± 1.63	2.0 ± 0.12
H314	17.4 ± 1.56	7.0 ± 0.58
H413	19.2 ± 1.47	2.0 ± 0.26

^a^Data are presented as mean of triplicate values ± SEM. IC_50_ values ≤20 *μ*g/mL are considered cytotoxic.

**Table 2:** The yield (mg) obtained and cytotoxicity (IC_50_) for fractions of *D. cinnabari* and subfractions DCc and DCd.

Fractions/subfractions	Yield (mg)	IC_50_ ± SEM (*μ*g/mL)^a^
DCa^b^	3.4	29.0 ± 0.64
DCb	3.0	>30
**DCc**	**7.0**	**5.8 ± 0.37**
**DCd**	**4.5**	**3.1 ± 0.13**
DCe	4.5	18.4 ± 0.76
DCf	3.2	>30
DCg	3.9	>30
DCc1^c^	2.0	>30
DCc2	1.0	25.4 ± 0.38
DCc3	1.0	5.1 ± 1.40
DCc4	1.6	13.1 ± 0.47
DCc5	1.7	25.3 ± 0.18
DCc6	1.4	>30
DCc7	0.9	12.4 ± 0.26
DCc8	0.9	11.8 ± 0.23
DCc9	1.4	5.4 ± 0.16
DCc10	1.9	6.1 ± 0.09
**DCc11**	**2.2**	**3.0 ± 0**
**DCc12**	**1.0**	**3.1 ± 0.04**
**DCc13**	**1.1**	**3.0 ± 0**
**DCc14**	**0.8**	**3.4 ± 0.07**
**DCc15**	**2.0**	**2.9 ± 0.07**
DCc16	1.3	4.2 ± 0.16
DCd1^d^	1.0	>30
DCd2	0.7	27.9 ± 0.63
DCd3	1.8	20.7 ± 0.77
DCd4	1.5	12.8 ± 0.12
DCd5	0.8	22.3 ± 1.16
DCd6	1.2	27.6 ± 0.57
DCd7	1.3	23.8 ± 0.33
DCd8	1.6	17.8 ± 1.62
DCd9	2.8	5.6 ± 0.12
**DCd10**	**1.0**	**3.9 ± 0.94**
**DCd11**	**2.0**	**3.1 ± 2.36**
**DCd12**	**2.0**	**3.2 ± 0.06**
**DCd13**	**1.5**	**4.0 ± 0.56**
DCd14	1.7	5.0 ± 0.12
**DCd15**	**2.4**	**3.3 ± 0.08**
**DCd16**	**2.0**	**2.9 ± 0.07**
**DCd17**	**2.0**	**3.9 ± 0.18**
DCd18	2.0	13.2 ± 0.13

^a^Data are presented as mean ± SEM in triplicate. Values in bold characters are considered cytotoxically active towards H400 cells (IC_50_ ≤ 10 *μ*g/mL for fractions; IC_50_ value ≤ 4 *μ*g/mL for subfractions).

^b^DCa, DCb,…, DCg: fractions of *D. cinnabari*.

^c^DCc1, DCc2,…, DCc16: subfractions isolated from fraction DCc.

^d^DCd1, DCd2,…, DCd18: subfractions isolated from fraction DCd.

**Table 3:** Cytotoxicity (IC_50_) against NHOF cells and selectivity index (SI) of selected active extract, fractions, and subfractions of *D. cinnabari*.

Selected compounds	IC_50_ ± SEM (*μ*g/mL)	SI
DC extract^a^	>30	ND
DCc^b^	>30	ND
DCd^b^	>30	ND
DCc15^c^	24 ± 2.77	8.28
DCd16^d^	>30	ND
Cisplatin	>30	ND

Data are presented as mean ± SEM in triplicate. Selectivity index is defined as the ratio of cytotoxicity (IC_50_ values) on normal human oral fibroblast (NHOF) cells to cancer cells (SI = IC_50_ NHOF cells/IC_50_ on H400 cells). SI values greater than 3 are considered to have high selectivity towards cancer cells.

ND: not detected (as the IC_50_ values on NHOF >30 *μ*g/mL).

^a^DC extract: methanol extract of *D. cinnabari*.

^b^DCc and DCd: fractions of *D. cinnabari*.

^c^DCc15: subfraction isolated from fraction DCc.

^d^DCd16: subfraction isolated from fraction DCd.

**Table 4:** Percentage of apoptotic cells upon treatments with studied compounds of *D. cinnabari* at concentration of IC_50_ for 24 hours.

Studied compounds of *D. cinnabari*	Percentage of apoptotic cells (%)
DC	58.14
DCc	67.03
DCd	74.43
DCc15	85.69
DCd16	41.99
Negative control	13.81

**Table 5:** Cell cycle analysis of H400 cells at 48 and 72 hours treated with studied compounds of *D. cinnabari* at concentration of IC_50_.

Compounds	Percentage of cells (%)			
	Sub-G1	G1	S	G2/M
48 hours				
Negative control	2.86 ± 0.27	63.52 ± 1.42	3.91 ± 0.05	17.11 ± 0.09
DC extract	27.87 ± 0.79^*∗*^	35.13 ± 0.62^*∗*^	5.21 ± 0.10^*∗*^	10.47 ± 0.17^*∗*^
DCc	29.78 ± 0.92^*∗*^	24.89 ± 0.43	5.90 ± 0.22^*∗*^	13.21 ± 0.36^*∗*^
DCd	30.28 ± 0.74^*∗*^	33.09 ± 0.50^*∗*^	5.47 ± 0.06^*∗*^	12.51 ± 0.22^*∗*^
DCc15	5.16 ± 0.23	62.31 ± 1.05^*∗*^	4.52 ± 0.06^*∗*^	13.37 ± 0.12^*∗*^
DCd16	26.33 ± 0.44^*∗*^	43.40 ± 1.14^*∗*^	6.93 ± 0.10^*∗*^	12.96 ± 0.17^*∗*^

72 hours				
Negative control	7.00 ± 2.40	88.04 ± 1.16	2.27 ± 0.60	2.66 ± 1.55
DC extract	64.2 ± 7.19^*∗*^	24.34 ± 2.71^*∗*^	7.95 ± 2.32^*∗*^	3.18 ± 2.38
DCc	56.45 ± 2.61^*∗*^	30.57 ± 7.90^*∗*^	8.08 ± 3.52^*∗*^	4.32 ± 3.40
DCd	59.8 ± 7.36^*∗*^	26.03 ± 3.06^*∗*^	10.18 ± 1.91^*∗*^	3.93 ± 2.48
DCc15	21.43 ± 7.59	70.69 ± 2.30^*∗*^	5.32 ± 3.71^*∗*^	2.28 ± 2.04
DCd16	68.43 ± 4.38^*∗*^	22.56 ± 2.52^*∗*^	7.46 ± 1.26^*∗*^	1.59 ± 0.93

The values are expressed as mean ± SEM in triplicate. ^*∗*^Significant difference compared to negative control with *p* < 0.05 through one-way ANOVA.

## References

[1] Nair S., Pillai M. R. (2005). Human papillomavirus and disease mechanisms: relevance to oral and cervical cancers. *Oral Diseases*.

[2] Casto B. C., Sharma S., Fisher J. L., Knobloch T. J., Agrawal A., Weghorst C. M. (2009). Oral cancer in Appalachia. *Journal of Health Care for the Poor and Underserved*.

[3] Wang B., Zhang S., Yue K., Wang X.-D. (2013). The recurrence and survival of oral squamous cell carcinoma: a report of 275 cases. *Chinese Journal of Cancer*.

[4] Cragg G. M., Newman D. J. (2005). Plants as a source of anti-cancer agents. *Journal of Ethnopharmacology*.

[5] Beutler J. A. (2009). Natural products as a foundation for drug discovery. *Current Protocols in Pharmacology*.

[6] Evans W. C. (2002). General methods associated with the phytochemical investigation of herbal products. *Trease and Evans Pharmacognosy*.

[7] Ramasamy S., Wahab N., Zainal Abidin N., Manickam S., Zakaria Z. (2012). Growth inhibition of human gynecologic and colon cancer cells by *Phyllanthus watsonii* through apoptosis induction. *PLoS ONE*.

[8] Gupta D., Bleakley B., Gupta R. K. (2007). Dragon's blood: botany, chemistry and therapeutic uses. *Journal of Ethnopharmacology*.

[9] Milburn M. (1984). Dragon's blood in east & west Africa, Arabia and the Canary Islands. *Africa: Rivista Trimestrale di Studi e Documentazione dell'Istituto Italiano per l'Africa e l'Oriente*.

[10] Machala M., Kubinova R., Horavova P., Suchy V. (2001). Chemoprotective potentials of homoisoflavonoids and chalcones of *Dracaena cinnabari*: modulations of drug-metabolizing enzymes and antioxidant activity. *Phytotherapy Research*.

[11] Mothana R. A. A., Lindequist U. (2005). Antimicrobial activity of some medicinal plants of the island Soqotra. *Journal of Ethnopharmacology*.

[12] Al-Fatimi M., Friedrich U., Jenett-Siems K. (2005). Cytotoxicity of plants used in traditional medicine in Yemen. *Fitoterapia*.

[13] Prime S. S., Nixon S. V. R., Crane I. J. (1990). The behaviour of human oral squamous cell carcinoma in cell culture. *The Journal of Pathology*.

[14] Mosmann T. (1983). Rapid colorimetric assay for cellular growth and survival: application to proliferation and cytotoxicity assays. *Journal of Immunological Methods*.

[15] Mahavorasirikul W., Viyanant V., Chaijaroenkul W., Itharat A., Na-Bangchang K. (2010). Cytotoxic activity of Thai medicinal plants against human cholangiocarcinoma, laryngeal and hepatocarcinoma cells in vitro. *BMC Complementary and Alternative Medicine*.

[16] Zhang G. H., Gurtu V., Kain S. R., Yan G. C. (1997). Early detection of apoptosis using a fluorescent conjugate of annexin V. *BioTechniques*.

[17] Boik J. (2001). *Natural Compounds in Cancer Therapy*.

[18] Ghareed M. A., Shoeb H. A., Madkour H. M. F., Refaey L. A. G. R., Mohamed M. A. M., Saad A. M. (2014). Antioxidant and cytotoxic activities of *Tectona grandis* linn leaves. *International Journal of Phytopharmacology*.

[19] Siti Syarifah M. M., Nurhanan M. Y., Muhd Haffiz J. (2011). Potential anticancer compound from *Cerbera odollam*. *Journal of Tropical Forest Science*.

[20] Juranek I., Suchy V., Stara D., Masterova I., Grancaiova Z. (1993). Antioxidative activity of homoisoflavonoids from *Muscari racemosum* and *Dracena cinnabari*. *Pharmazie*.

[21] Body M. R., Paull K. D. (1995). Some practical considerations and applications of the national cancer institute in vitro anticancer drug discovery screen. *Drug Development Research*.

[22] Taraphdar A. K., Roy M., Bhattacharya R. K. (2001). Natural products as inducers of apoptosis: implication for cancer therapy and prevention. *Current Science*.

[23] Kerr J. F., Wyllie A. H., Currie A. R. (1972). Apoptosis: a basic biological phenomenon with wide-ranging implications in tissue kinetics. *British Journal of Cancer*.

[24] Hengartner M. O. (2001). Apoptosis: corralling the corpses. *Cell*.

[25] Baskić D., Popović S., Ristić P., Arsenijević N. N. (2006). Analysis of cycloheximide-induced apoptosis in human leukocytes: fluorescence microscopy using annexin V/propidium iodide versus acridin orange/ethidium bromide. *Cell Biology International*.

[26] Boutonnat J., Barbier M., Muirhead K., Mousseau M., Ronot X., Seigneurin D. (1999). Optimized fluorescent probe combinations for evaluation of proliferation and necrosis in anthracycline-treated leukaemic cell lines. *Cell Proliferation*.

[27] Jurisicova A., Varmuza S., Casper R. F. (1996). Programmed cell death and human embryo fragmentation. *Molecular Human Reproduction*.

[28] Roy C., Brown D. L., Little J. E. (1992). The topoisomerase II inhibitor teniposide (VM-26) induces apoptosis in unstimulated mature murine lymphocytes. *Experimental Cell Research*.

[29] Tomei L. D., Shapiro J. P., Cope F. O. (1993). Apoptosis in C3H/10T1/2 mouse embryonic cells: evidence for internucleosomal DNA modification in the absence of double-strand cleavage. *Proceedings of the National Academy of Sciences of the United States of America*.

[30] Gooch J. L., Yee D. (1999). Strain-specific differences in formation of apoptotic DNA ladders in MCF-7 breast cancer cells. *Cancer Letters*.

[31] Johnson V. L., Ko S. C. W., Holmstrom T. H., Eriksson J. E., Chow S. C. (2000). Effector caspases are dispensable for the early nuclear morphological changes during chemical-induced apoptosis. *Journal of Cell Science*.

[32] Yazan L. S. (2003). *Cytotoxic properties of anthraquinones (nordamnacanthal and damnacanthal) from roots of Morinda elliptica [Ph.D. thesis]*.

[33] Jiang L., Luo M., Liu D. (2013). BAD overexpression inhibits cell growth and induces apoptosis via mitochondrial-dependent pathway in non-small cell lung cancer. *Cancer Cell International*.

[34] Perry S. W., Norman J. P., Barbieri J., Brown E. B., Gelbard H. A. (2011). Mitochondrial membrane potential probes and the proton gradient: a practical usage guide. *BioTechniques*.

[35] Simon H.-U., Haj-Yehia A., Levi-Schaffer F. (2000). Role of reactive oxygen species (ROS) in apoptosis induction. *Apoptosis*.

[36] Kang M. H., Reynolds C. P. (2009). BcI-2 Inhibitors: targeting mitochondrial apoptotic pathways in cancer therapy. *Clinical Cancer Research*.

[37] Ajenjo N., Canoñón E., Sánchez-Pérez I. (2004). Subcellular localization determines the protective effects of activated ERK2 against distinct apoptogenic stimuli in myeloid leukemia cells. *The Journal of Biological Chemistry*.

[38] Kang M. H., Reynolds C. P. (2009). Bcl-2 Inhibitors: targeting mitochondrial apoptotic pathways in cancer therapy. *Clinical Cancer Research*.

[39] Kuwana T., Newmeyer D. D. (2003). Bcl-2-family proteins and the role of mitochondria in apoptosis. *Current Opinion in Cell Biology*.

[40] Adams J. M., Cory S. (2007). The Bcl-2 apoptotic switch in cancer development and therapy. *Oncogene*.

[41] Sommer P., Cowen R. L., Berry A. (2010). Glucocorticoid receptor over-expression promotes human small cell lung cancer apoptosis in vivo and thereby slows tumor growth. *Endocrine-Related Cancer*.

[42] Gogvadze V., Orrenius S., Zhivotovsky B. (2006). Multiple pathways of cytochrome c release from mitochondria in apoptosis. *Biochimica et Biophysica Acta—Bioenergetics*.

[43] Tait S. W. G., Green D. R. (2010). Mitochondria and cell death: outer membrane permeabilization and beyond. *Nature Reviews Molecular Cell Biology*.

[44] Lewin B., Cassimeris L., Lingappa V. R. (2007). *Cell*.

[45] Alberts B., Johnson A., Lewis J., Raff M., Roberts K., Walter P. (2008). *Molecular Biology of the Cell*.

[46] Harada H., Yamashita U., Kurihara H., Fukushi E., Kawabata J., Kamei Y. (2002). Antitumor activity of palmitic acid found as a selective cytotoxic substance in a marine red alga. *Anticancer Research*.

[47] Hu X., Zhang X., Qiu S., Yu D., Lin S. (2010). Salidroside induces cell-cycle arrest and apoptosis in human breast cancer cells. *Biochemical and Biophysical Research Communications*.

